# Discomfort of postoperative patients with aortic dissection after discharge: telephone follow-up analysis of a cross-sectional study

**DOI:** 10.1186/s13019-022-01779-w

**Published:** 2022-03-09

**Authors:** Xiaorong Lang, Sufang Huang, Quan Wang, Danni Feng, Yaru Xiao, Miqi Li, Zhiran Guo, Quan Zhou

**Affiliations:** 1 School of Nursing, Tongji Medical College of Huazhong University of Science and Technology, Wuhan, China; 2 Tongji Hospital Affiliated to Tongji Medical College of Huazhong University of Science and Technology, No. 1095, Jiefang Avenue, Wuhan, Hubei Province China

**Keywords:** Aortic dissection, Postoperative discomfort, Follow-up

## Abstract

**Background:**

Postoperative discomfort is one of the important manifestations of disease changes, but few studies have reported detailed description of postoperative discomfort in patients with aortic dissection after discharge. The aim of this study is to investigate the discomfort symptoms and to explore the possible influencing factors of discomfort symptoms.

**Method:**

This cross-sectional study based on convenience sampling collected medical records from 999 patients hospitalized in Tongji Hospital Affiliated to Tongji Medical College of Huazhong University of science and technology, Wuhan, Hubei, China from January 1, 2019 to December 31, 2019. Postoperative patients with first onset and confirmed aortic dissection were eligible for follow-up. Telephone follow-up was conducted from July 20, 2020 to August 20, 2020. Symptoms of discomfort were reported by patients or their immediate family members. Univariate and multivariable logistic regression analysis were performed to identify factors associated with symptoms of discomfort.

**Results:**

A total of 675 patients were followed up, 185 patients (27.4%) were lost to follow-up, and the remaining 490 patients were divided into survival group (N = 428) and death group (N = 62) and were included in the study. There was no difference in gender and age among the three groups. 152 of 428 patients reported discomfort. The uncomfortable symptoms of postoperative patients were diverse, and mainly manifested as back and chest pain (32.24%, 49/152), chest tightness (15.79%, 24/152), dizzy (10.53%, 16/152) and weakness (10.53%, 16/152). Multivariable logistic regression analysis of postoperative discomfort showed length of discharge (OR 0.995; P 0.018; 95% CI 0.990–0.999) and positive history of drinking (OR 3.519; P 0.018; 95% CI 1.236–10.022) were significant among patients with Stanford A AD, and diagnosis was made in the first visiting hospital (OR 0.395; P 0.001; 95% CI 0.230–0.677) was a protective factor for patients.

**Conclusions:**

The incidence of postoperative discomfort in patients with aortic dissection was high and the symptoms were diverse and not single. In order to reduce the possibility of postoperative discomfort, it is important to formulate effective public policies to limit the public to drink alcohol and timely diagnose aortic dissection. Long term follow-up is necessary for patients with aortic dissection to observe the recovery process of aortic dissection.

## Background

Aortic dissection (AD) is an emergent and life-threatening disorder. A number of studies have reported high mortality of AD in a short time, and the mortality rate can be as high as 35% when there are other serious complications [1–3]. With the passage of time, the treatment of AD has experienced the transition from open repair to endovascular treatment [4], and the number of patients who underwent surgical intervention has increased significantly [5]. Although the survival rate of patients with AD has been improved due to the improvement of surgical treatment, patients with AD still face physical and psychological problems, continuing for months or years [6].

From the perspective of bio-psychosocial medicine model, human health and disease are not only biological processes, but also closely related to psychology and society and exert influence on people together [7]. When people feel uncomfortable subjectively, it not only means that they may suffer from a certain disease, but also means that their physical function and psychological state are negatively affected, leading to decline in quality of life. Although heart surgery has been concerned as a life-threatening event for patients and associated with a strong encroachment on the person’s body and integrity [8, 9], most clinical studies have only focused on postoperative complications instead of their subjective symptoms. Numerous studies have concluded that discomfort symptom could be an important determinant of people’s life, as patients with chronic pain might be unable to work or fall into disability [10, 11]. Meanwhile, patients with physical complaints would be at risk of developing psychological distress [12], and further lead to adverse cardiovascular events [13]. However, there seems to be little attention paid to the detailed description of discomfort symptoms in patients with AD after discharge.

The aim of this study is to understand the incidence and characteristics of postoperative discomfort symptoms, and explore determinants of discomfort symptoms, so as to help clinical treatment decision-making and help patients achieve a higher quality of life after surgery.

## Methods

### Study design

This was a cross-sectional study. We retrospectively collected data for all participants from a single tertiary referral hospital, Tongji Hospital Affiliated to Tongji Medical College of Huazhong University of science and technology, Wuhan, Hubei, China. Data were anonymous and compiled from the hospital electronic medical record (EMR) system. Due to the influence of COVID-19, we conducted telephone follow-up from July 20, 2020 to August 20, 2020. By reviewing the related literature, we determined the content of data collection and follow-up. Participants were selected by convenience sampling.

### Participants

The target sample size of participants was determined using the formula N = Zα^2^P (1 − P)/d^2^, in which α = 0.05 and Zα = 1.96, and the estimated acceptable margin of error for proportion d was 0.05. The proportion of discomfort was estimated at 26.7%, based on a previous study of patients with abdominal aortic aneurysm after open repair [14]. Considering the unavoidable loss to follow-up, we expanded the sample size by 30%, and the number of patients included in the analysis should be no less than 391 at last. Based on the calculation result of sample size, patients admitted to the hospital from January 1, 2019 to December 31, 2019 were investigated.

Inclusion criteria: (1) Diagnosed AD; (2) first hospitalization; (3) had surgery; (4) age ≥ 18.

Exclusion criteria: (1) lost to follow-up; (2) infected COVID-19.

### Data collection

Demographic variables and data related to disease diagnosis and treatment were collected. Demographic variables included age, sex, education level, marital status, place of residence, smoking, drinking and history of hypertension. Data related to disease diagnosis and treatment included types of AD, types of operations, blood pressure and heart rate at admission, length of stay and discharge and whether it was diagnosed in the first visiting hospital.

Whether the patient had any discomfort symptoms was obtained by following a constructive questionnaire. The patient's death after operation was based on EMR and follow-up results, and the time of death outside the hospital was subject to the report of the patient's immediate family.

**Fig. 1:**
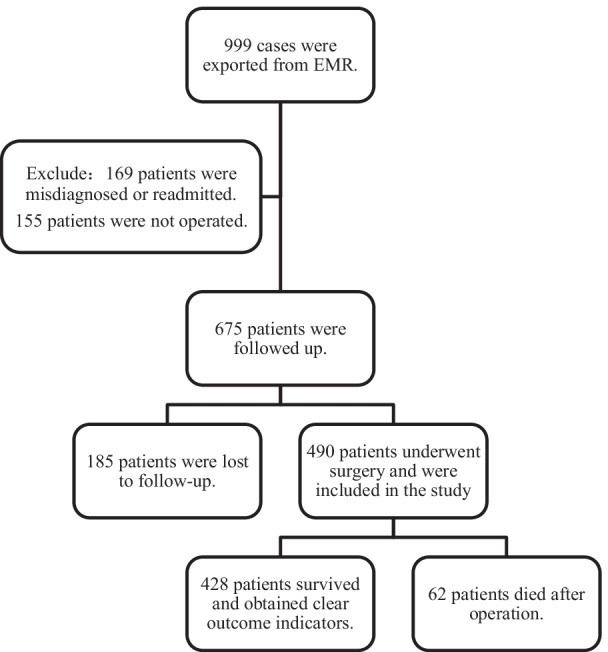
Flow chart of patients enrollment

All the 6 researchers involved in the follow-up work were nursing staff, and they had received relevant knowledge training on aortic dissection before the work to ensure that they could give the appropriate and consistent guidance during the follow-up process. In order to ensure the quality of follow-up, after getting the answers to the corresponding questions, the response should be repeated to confirm whether the recorded information was accurate. The identification rules of lost follow-up were as follows: (1) The phone had shut down, or there were at least two unanswered calls in different three days, with an interval of more than two hours; (2) The answers were unwilling to cooperate with the interview, hung up the phone and no longer answered the phone later; (3) The phone number was an invalid number; (4)There was no clear answer about outcome indicators. Patients screening process is detailed in Fig. 1.

The follow-up results were recorded in Excel according to the template, and were collated and checked by two people after the end.

### Outcomes

The main outcomes were the incidence and symptoms of discomfort. Discomfort was defined as the subjective pain experience or abnormal feeling that existed continuously or intermittently from discharge to telephone follow-up.

### Statistical analyses

SPSS v22.0 software was used to describe the basic data of patients. Categorical variables were presented as numbers and percentages while continuous variables were reported as means and standard deviation (SD). Continuous variables were supplemented by mean interpolation. Except education level, marital status and place of residence, all variables were put into single-factor regression analysis first and variables with a P value < 0.1 were put into multifactor regression equation. The results of multivariable logistic regression analysis were significant when P value was less than 0.05. Odds ratio (OR) and 95% confidence interval (CI) were also calculated. Due to the differences in pathophysiology between type A and type B AD, the influencing factors of postoperative discomfort were analyzed respectively. However, because of the psychological resistance of the family members of the dead patients, we failed to obtain the relatively complete information of the dead patients, so the patients who died after operation were not included in the analysis of the causes.

## Results

### Basic characteristics of research objects

Based on the inclusion and exclusion criteria, 675 patients were followed up. As a result, 185 (27.4%, 185/675) patients were lost to follow up, and a total of 490 participants were selected for final data analysis (see for Fig. 1 a flow chart). There was no significant difference in age and gender between patients who were lost to follow-up and patients included in this study. In our study, patients with AD were mainly male and the mean age of patients was about 55 years old. Endovascular aortic repair (EVAR) was the main surgical treatment. The main type of AD was Stanford B (82.1%) in survival group, while death group was Stanford A (64.5%). The main treatment of survival group was EVAR. In the death group, open repair was more often used. Other characteristics of the patients were shown in Table 1.

**Table 1:** Baseline characteristics of the patients (N = 675)

Variable	Survival (N = 428)	Death (N = 62)	Loss to follow up (N = 185)	*P* value
Age, years, SD	55.9 (11.2)	54.8 (12.1)	56.4 (13.4)	0.676
*Sex*				0.237
Male	353 (82.5)	56 (90.3)	150 (81.1)	
Female	75 (17.5)	6 (9.7)	35 (18.9)	
*Education level*				–
Illiteracy	32 (7.5)	2 (3.2)	13 (7.0)	
Primary school	91 (21.3)	14 (22.6)	33 (17.8)	
Junior high school	136 (31.8)	16 (25.8)	66 (35.7)	
High school	96 (22.4)	16 (25.8)	40 (21.6)	
Junior college or above	65 (15.2)	13 (21.0)	24 (13.0)	
Unknown	8 (1.9)	1 (1.6)	9 (4.9)	
*Marital status*				–
Unmarried	5 (1.2)	1 (1.6)2	5 (3.3)	
Married	415 (97.0)	58 (93.5)	176 (95.1)	
Divorce	2 (0.5)	1 (1.6)	1 (1.6)	
Widowed	6 (1.4)	0 (0.0)	0 (0.0)	
Unknown	0 (0.0)	2 (3.2)	3 (1.6)	
*Place of residence*				–
City	116 (27.1)	28 (45.2)	66 (35.7)	
Town	129 (30.1)	6 (9.7)	59 (31.9)	
Countryside	183 (42.8)	28 (45.2)	58 (31.4)	
Unknown	0 (0.0)	0 (0.0)	2 (1.1)	
*Smoking*				0.000*
Yes	178 (41.6)	13 (21.0)	51 (27.6)	
No	250 (58.4)	49 (79.0)	134 (72.4)	
*Drinking*				0.006*
Yes	163 (38.1)	12 (19.4)	59 (31.9)	
No	265 (61.9)	50 (80.6)	126 (68.1)	
*Was there a history of hypertension?*				
Yes	259 (61.5)	26 (41.9)	99 (53.5)	0.006*
No	162 (38.5)	36 (58.1)	86 (46.5)	
*Types of aortic dissection*				
Stanford A	79 (18.5)	40 (64.5)	40 (21.6)	0.000*
Stanford B	349 (81.5)	22 (35.5)	145 (78.4)	
*Operation methods*				0.000*
EVAR	367 (85.7)	23 (37.1)	146 (78.9)	
Open repair	61 (14.3)	39 (62.9)	39 (21.1)	
SBP, mmHg, SDs	146.8 (28.1)	137.1 (34.3)	138.7 (26.3)	0.001*
DBP, mmHg, SDs	82.6 (15.1)	76.5 (18.0)	78.7 (15.5)	0.001*
HR, SD	80.1 (14.4)	85.7 (21.1)	81.1 (14.9)	0.084
Length of stay, days, SD	13.5 (9.1)	13.5 (11.5)	13.2 (13.7)	0.320
Length of discharge, days, SD	407.1 (114.6)	–	–	–
*Diagnosis made in the first visiting hospital*				–
Yes	341 (79.9)	–	–	–
No	87 (20.3)	–	–	–

### Characteristics of discomfort symptoms of discomfort

**Fig. 2:**
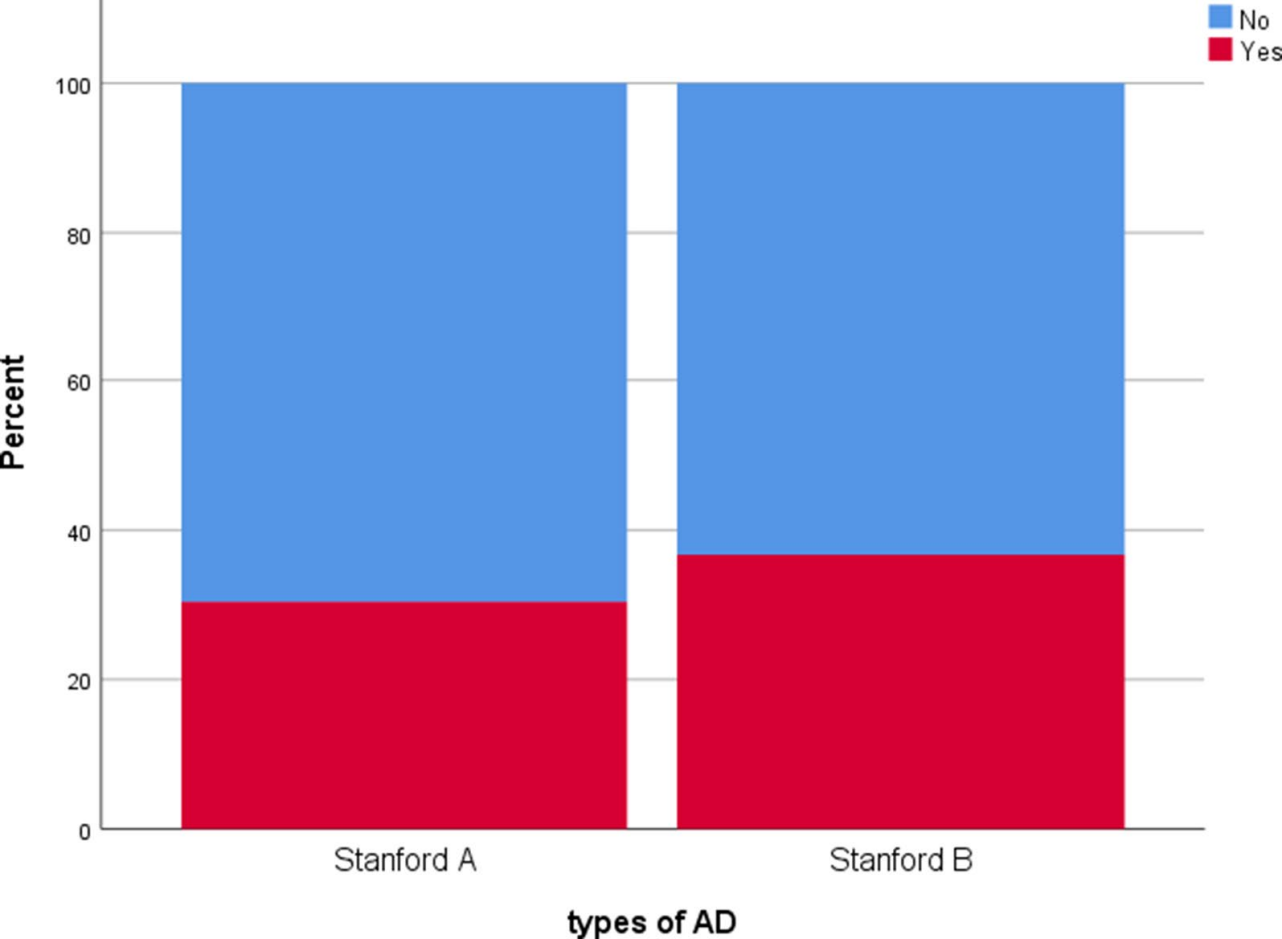
the incidence of postoperative discomfort in Stanford A and Stanford B

**Fig. 3:**
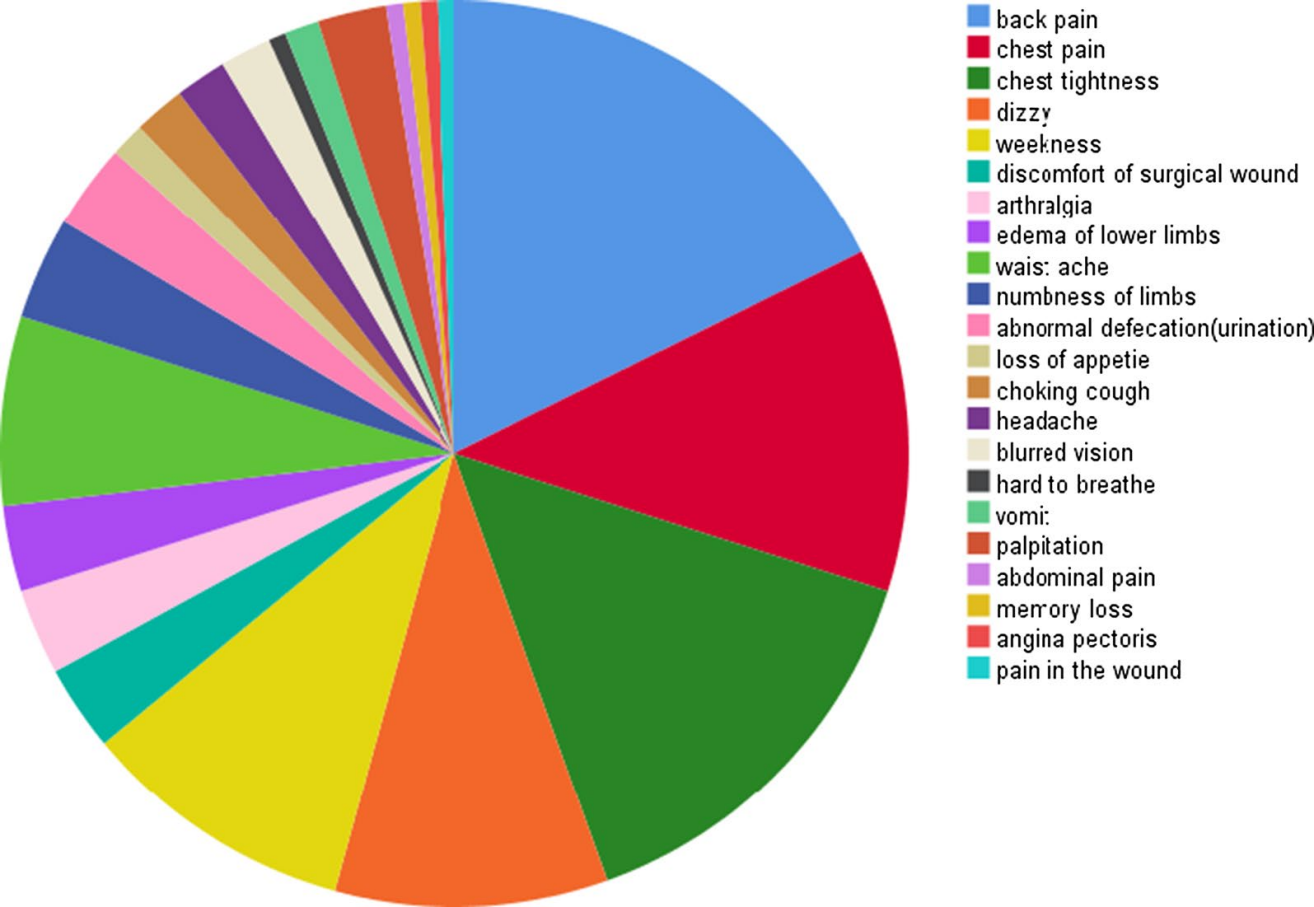
Proportion diagram of discomfort symptoms

According to Fig. 2, a total of 152 patients reported different symptoms of discomfort, including 24 with Stanford A and 128 with Stanford B. As shown in Fig. 3, the main symptoms of discomfort were back and chest pain (32.24%, 49/152), chest tightness (15.79%, 24/152), dizzy (10.53%, 16/152) and weakness (10.53%, 16/152). The discomfort symptoms of patients showed a trend of diversification. Some patients (9.2%, 14/152) had two or more kinds of discomfort, showing the characteristics of non-singleness.

### Determinants of discomfort

According to Table 2, the incidence of discomfort was significantly increased on multivariable analysis, when the patient was positive for alcohol drinking history (OR, 3.519; P, 0.018; 95% CI 1.236–10.022). However, length of discharge was associated with a reduced incidence of discomfort (OR, 0.995; P, 0.018; 95% CI 0.990–0.999) on multivariate logistic regression analysis. In Table 3, multivariable logistic regression analysis showed that the diagnosis made in the first visiting hospital was the protective factor of discomfort for patients with type B AD (OR, 0.395; P, 0.001; 95%CI, 0.230–0.677). The more timely the diagnosis was, the less likely the patient was to develop discomfort.

**Table 2:** Factors in association with postoperative discomfort of patients with Stanford A AD

Variables	Univariate analyses		Multivariate analysis		
	OR (95% CI)	P value	β	OR (95% CI)	P value
Age	0.991 (0.943, 1.042)	0.991	–	–	–
Sex (male)	1.345 (0.398, 4.542)	0.633	–	–	–
Smoking (yes)	0.519 (0.191, 1.409)	0.198	–	–	–
Drinking (yes)	3.412 (1.257, 9.263)	0.016*	1.258	3.519 (1.236, 10.022)	0.018*
History of hypertension (positive)	1.282 (0.453, 3.630)	0.640	–	–	–
Operation methods (open repair)	1.181 (0.416, 3.359)	0.754	–	–	–
SBP	0.995 (0.979, 1.011)	0.555	–	–	–
DBP	1.001 (0.970, 1.033)	0.946	–	–	–
HR	0.994 (0.965, 1.024)	0.700	–	–	–
Length of stay	1.017 (0.971, 1.065)	0.468	–	–	–
Length of discharge	0.995 (0.990, 0.999)	0.016*	− 0.005	0.995 (0.990, 0.999)	0.018*
Diagnosis made in the first visiting hospital (yes)	1.176 (0.367, 3.771)	0.785	–	–	–

**Table 3:** Factors in association with postoperative discomfort of patients with Stanford B AD

Variables	Univariate analyses		Multivariate analysis		
	OR (95% CI)	*P* value	β	OR (95% CIs)	*P* value
Age	0.994 (0.975, 1.013)	0.523	–	–	–
Sex (male)	1.355 (0.772, 2.376)	0.290	–	–	–
Smoking (yes)	0.717 (0.461, 1.115)	0.139	–	–	–
Drinking (yes)	1.066 (0.681, 1.667)	0.780	–	–	–
History of hypertension (positive)	1.124 (0.718, 1.758)	0.609	–	–	–
Operation methods (open repair)	0.686 (0.131, 3.587)	0.655	–	–	–
SBP	0.997 (0.989, 1.005)	0.505	–	–	–
DBP	0.997 (0.982, 1.012)	0.660	–	–	–
HR	1.004 (0.989, 1.020)	0.608	–	–	–
Length of stay	1.027 (0.998, 1.057)	0.070*	0.024	1.025 (0.995, 1.055)	0.106
Length of discharge	0.999 (0.997, 1.001)	0.220	–	–	–
Diagnosis made in the first visiting hospital (yes)	0.386 (0.226, 0.661)	0.001*	− 0.930	0.395 (0.230, 0.677)	0.001*

## Discussion

This was the first quantitative study to investigate postoperative discomfort in patients with AD and to analyze the influencing factors. According to the relevant survey, the prevalence of aortic dissection was higher in men and it occurred in people aged 40–60 [4, 15], and with the development of medical technology, EVAR has become the main treatment for AD, while was consistent with our results. However, the mortality rate for Stanford A AD reported in Table 1 was higher than in other studies, which probably because it took some time for patients to be referred to our hospital for treatment instead of having surgery immediately even after diagnosis due to weak public health network, which meant further progression of the disease with high mortality [16].

Discomfort was common in postoperative patients with disease of artery. Carlijn et al. found that all participants had physical complaints such as thoracic pain, dyspnea or tachycardia in an in-depth interview with 11 patients with TAD who had experienced aortic surgery [12]. A study described that four of 15 patients with experience of open repair of abdominal aortic aneurysm reported experiencing remaining physical complications at home after surgery, such as lacking appetite, experiencing altered taste sensations, feeling overwhelming fatigue [14]. In our study, the incidence of self-reported postoperative discomfort was 35.5%, the main symptoms were pain, chest tightness, dizzy and weakness, which was similar to other studies. However, our findings suggested that the discomfort of postoperative patients were varied and some patients had multiple discomforts at the same time. Furthermore, all symptoms of discomfort reported by the patient were listed in detail to help with a fuller understanding, which had rarely been reported before. Discomfort reflected the patients' physical recovery [17]. For postoperative patients, recovery was still a challenging process, and discomfort symptoms limited their activities, which reduced the sense of participation in daily life and caused psychological burden. Therefore, more attention should be paid to the postoperative discomfort and recovery of patients with AD, and continuous interdisciplinary cooperative medical care for patients with aortic dissection is necessary.

The recovery of AD is a long-term process. Discharge time of patients included in this study ranged from 7 to 19 months. For patients with Stanford A AD, they experienced greater surgical trauma and needed more time to recover, which might explain the presence of discomfort depended on the length of discharge. A follow-up of an average of 108 months found that heath of patients with AD gradually improved over time [18]. Therefore, we need to carry out long-term follow-up to observe whether the patient's discomfort symptoms will relief or disappear. Furthermore, positive history of drinking was also one of the reasons for discomfort, which might be caused by the toxic effect of alcohol or its metabolites resulting in myocardial damage and larger diameters and lower distensibilities of alcohol consumption [19]. Although there were some studies showing that moderate drinking could reduce the risk of cardiovascular disease [20, 21], the relationship between alcohol consumption and AD is not so clear, and alcohol consumption remains a risk factor in patients with AD that needs to be valued and controlled.

Another finding in our study was that patients with Stanford B AD who could not be diagnosed as early as possible were more likely to suffer from postoperative discomfort. In China, the first diagnosis and treatment of AD is mostly completed in primary hospitals or health care centers, which do not have the ability of diagnosis due to the weakness of public health network [16]. Previous hospital-based studies from specialized centers or studies from retrospective registry data indicated that the delay from onset of symptoms to diagnosis could range from 4.3 h to more than 24 h, and delayed diagnosis means patients receive treatment for AD more lately [16, 23]. It was confirmed that delayed diagnosis might lead to devastating outcomes (amputations or renal failure) even death [25]. And our findings supplemented a neglected result that delayed diagnosis could lead to physical discomfort of patients after surgery, which may have a negative impact on patients. Therefore, paying attention to the improvement of the quality and efficiency of diagnosis and treatment can not only reduce the likelihood of serious consequences, but also help reduce the occurrence of postoperative adverse events. Unfortunately, due to the lack of pre-diagnosis data, our study could not determine the specific time of diagnosis.

### Limitations

This study has several limitations. First, due to the lack of evaluation tools for the symptoms of patients with AD after discharge, the discomfort of patients was reported by themselves, which might have some subjective bias. Therefore, it is important to develop evaluation tools for the discomfort symptoms of postoperative patients with AD in the next piece of research. Second, this was a single bus, cross-sectional survey, patients were discharged from hospital from 7 to 19 months at the time of follow-up, this study did not represent the long-term prognosis and outcome of patients with AD, so further follow-up is needed to improve the results and more multi-center and cohort studies are needed in the future. Third, although the follow-up rate of this study was 72.6%, follow-up bias may still exist. Finally, we explored and analyzed the causes of discomfort in type A AD. Although the sample size was small, the results were meaningful, and a larger sample size was needed in the future to clarify the conclusions.

## Conclusion

Our study indicated that patients with AD had a higher proportion of postoperative discomfort symptoms. And through the detailed description of postoperative discomfort symptoms found that discomfort symptoms were varied and may not be singular, which meant there were still problems with the recovery of patients. Therefore, medical workers should pay attention to the physical condition of patients after operation and give early targeted guidance to prevent serious consequences. And how to make patients diagnosed as soon as possible and guide sensible drinking will still be important problems that medical workers need to solve. However, long-term follow-up is required to monitor the patients' recovery.

## Data Availability

The data that support the findings of this study are available on reasonable request from the corresponding author. The data are not publicly available due to privacy or ethical restrictions.

## Abbreviations

AD: Aortic dissection
EMR: Electronic medical record
EVAR: Endovascular aortic repair
SBP: Systolic blood pressure
DBP: Diastolic blood pressure
HR: Heart rate
OR: Odds ratio
CI: Confidence interval
